# Ferlins and TgDOC2 in *Toxoplasma* Microneme, Rhoptry and Dense Granule Secretion

**DOI:** 10.3390/life11030217

**Published:** 2021-03-09

**Authors:** Daniel N. A. Tagoe, Allison A. Drozda, Julia A. Falco, Tyler J. Bechtel, Eranthie Weerapana, Marc-Jan Gubbels

**Affiliations:** 1 Department of Biology, Boston College, Chestnut Hill, MA 02467, USA; dnatagoe@gmail.com (D.N.A.T.); drozda@bc.edu (A.A.D.); 2 Department of Chemistry, Boston College, Chestnut Hill, MA 02467, USA; falcoju@bc.edu (J.A.F.); tylerbechtel@gmail.com (T.J.B.); eranthie@bc.edu (E.W.)

**Keywords:** Apicomplexa, *Toxoplasma*, microneme, rhoptry, dense granule, exocytosis, DOC2

## Abstract

The host cell invasion process of apicomplexan parasites like *Toxoplasma gondii* is facilitated by sequential exocytosis of the microneme, rhoptry and dense granule organelles. Exocytosis is facilitated by a double C2 domain (DOC2) protein family. This class of C2 domains is derived from an ancestral calcium (Ca^2+^) binding archetype, although this feature is optional in extant C2 domains. DOC2 domains provide combinatorial power to the C2 domain, which is further enhanced in ferlins that harbor 5–7 C2 domains. Ca^2+^ conditionally engages the C2 domain with lipids, membranes, and/or proteins to facilitating vesicular trafficking and membrane fusion. The widely conserved *T. gondii* ferlins 1 (FER1) and 2 (FER2) are responsible for microneme and rhoptry exocytosis, respectively, whereas an unconventional TgDOC2 is essential for microneme exocytosis. The general role of ferlins in endolysosmal pathways is consistent with the repurposed apicomplexan endosomal pathways in lineage specific secretory organelles. Ferlins can facilitate membrane fusion without SNAREs, again pertinent to the Apicomplexa. How temporal raises in Ca^2+^ combined with spatiotemporally available membrane lipids and post-translational modifications mesh to facilitate sequential exocytosis events is discussed. In addition, new data on cross-talk between secretion events together with the identification of a new microneme protein, MIC21, is presented.

## 1. Introduction

**Figure 1:**
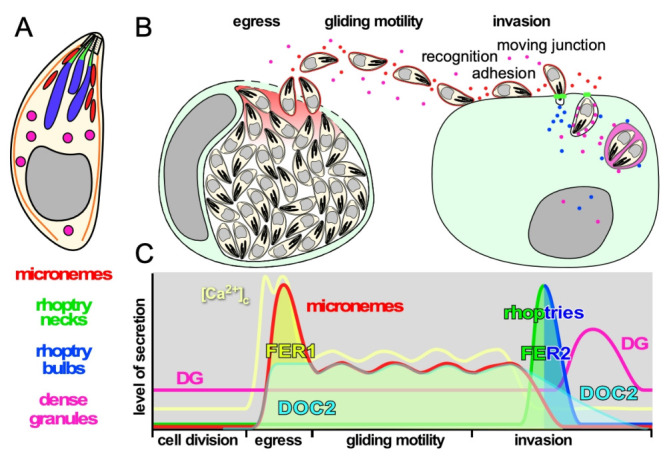
Schematic representation of the function and timing of secretion events in the *T. gondii* lytic cycle. (**A**). Schematic representation of *T. gondii* and its three secretory organelles, serving as legend for the other panels. (**B**). Schematic representation of secretion events throughout the lytic cycle. (**C**). Schematic representation of the levels of secretion of each organelle and the levels of intracellular calcium [Ca^2+^]_c_ corresponding with the secretion events throughout the lytic cycle. Timeline on the *X*-axis corresponds with the events in panel B. Note that the invasion process completes in less than a minute and is shown in a stretched-out time window. The two spikes in [Ca^2+^]_c_ at the time of egress correspond with the release of intracellular stores followed by the influx of extracellular Ca^2+^, while the [Ca^2+^]_c_ oscillates during gliding in concert with bursts of motility [4]. The activity of DOC2 and FER1 and FER2 is also indicated.

The opportunistic apicomplexan parasite *Toxoplasma gondii* causes significant disease in people with immature or compromised immune systems [1]. Central to the pathogenesis of all apicomplexan parasites, including malaria causing *Plasmodium falciparum*, is the rapid serial completion of lytic replication rounds causing tissue damage and inflammation. Completion of the lytic cycle requires host cell invasion and egress, which are mediated by sequential exocytosis of, respectively, the apically located micronemes and rhoptries and the more scattered dense granules organelles [2,3] (Figure 1A,B). 

Microneme protein function ranges from pore-forming protein (PLP1) facilitating egress, to a spectrum of adhesion molecules enabling the gliding motility and invasion machinery that mature through the action of microneme secreted-serine proteases (subtilisins) [5,6]. Microneme secretion is dosed as long as parasites are between host cells, a necessity to support sustained gliding motility, which also powers host cell invasion. Upon contact with an appropriate host cell the parasite injects the contents of its rhoptry organelles directly into the host cell cytosol. Rhoptry neck proteins (RONs) residing closest to the site of ejection, are released first to establish the moving junction, which is critical to efficient invasion. The rhoptry bulb proteins (ROPs) are released in the same secretion event (Figure 1B,C), and subvert a variety of host cell pathways to secure parasite replication and survival [7,8,9]. In addition, the rhoptries contain cholesterol and phosphatidylcholine presumably required to establish the parasitophorous vacuole membrane [10,11]. Upon abortion of an invasion event, rhoptries are still secreted as ‘evacuoles’ into noninvaded host cells and enable manipulation of these host cells similar to invaded host cells [12,13]. Dense granule proteins (GRA) are lastly secreted after PVM formation into the PV lumen from where they traffic to the PVM, intravacuolar network (IVN), host cell cytoplasm and even to the nucleus [14]. Dense granules with non-overlapping contents have been described, although it is not known whether these have different functions and/or different secretion kinetics [15]. GRA proteins have been implicated in several functions from interference with host cell signaling [16], transfer of small molecules between host cells and PV [17], manipulation of host cell immune response [18] and even parasite egress [19]. 

C2 domains are composed of 80–160 amino-acids that fold into β-sheets with specific binding characteristics that are found in a large and diverse set of eukaryotic proteins that can be grouped into several families [20]. Most C2 domains have lipid binding characteristics that are family specific [21], but they can also interact with other proteins [22,23,24]. The most diverse C2 domain protein family is derived from a protein kinase C (PKC) archetype that is unique among C2 domains in that it can stabilize Ca^2+^ ions (up to 3 per C2 domain) in a pocket composed of three loops emanating from the β-sheets. This family almost exclusively functions in signal transduction, membrane trafficking and fusion through conditionally engaging lipids and/or membranes upon changes in [Ca^2+^]_c_. However, not all members have retained the Ca^2+^-binding capacity and their membrane association could be conditional upon the presence of lipids in certain membranes. In principle, these C2 domain have two different lipid or membrane binding sites, which are not variably conserved: one in the Ca^2+^-binding loop region, and one in a lateral concavity in the β sheets [20].

**Figure 2:**
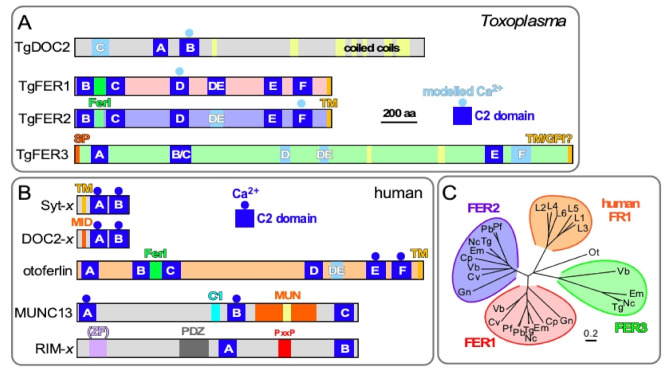
Comparative topologies of Ca^2+^-mediated exocytosis machinery in *Toxoplasma* and humans and ferlin phylogeny. (**A**). *Toxoplasma* encodes four DOC2 domain proteins: TgDOC2 and three ferlin proteins. Ferlins are defined by 5 to 7 C2 domains. TgFER3 contains an N-terminal signal peptide (SP), which, in combination with the C-terminal TM domain, could signal GPI-anchor addition at the C-terminus. Yellow shades in TgFER3 represent coiled-coil domains. Light blue C2 domains are degenerate (defined as having a *p*-value below the cut-off in PFam database searches); light blue shaded Ca^2+^ binding to C2 is based on modeling. The C2 domains labeled A–F by established conventions [28,30,42]. TM: C-terminal transmembrane domain; FerI: conserved ferlin domain of unknown function. Modified from [43]. (**B**). The spectrum of types of C2 domain containing proteins in humans with roles in Ca^2+^-dependent secretion. Calcium sensors are present in the Syt-x, DOC-x, and ferlin (otoferlin shown as representative) families, though not all family members are Ca^2+^ sensors. MID = MUN Interaction Domain. MUN = Mammalian Uncoordinated domain. ZF = Zn Finger; PxxP = Pro-rich. (**C**). Phylogenetic analysis of apicomplexan, chromerid and human ferlins. The following abbreviations are used: human ferlins “L1-L5” FR1L1-5 (FR1L1 (dysferlin; O75923.1), FR1L2 (otoferlin; Q9HC10.3), FR1L3 (myoferlin; Q9NZM1.1), FR1L4 (A9Z1Z3.1), FR1L5 (A0AVI2.2), FR1L6 (Q2WGJ9.2)), Ot: green algae *Ostreococcus tauri* (Q01FJ7); Chromerids: “Vb” *Vitrella brassicaformis* (VbFER1 (Vbre_12074 + Vbra_12075), VbFER2 (Vbra_9198)) and “Cv” *Chromera velia* (CvFER1 (Cvel_17519.2) and CvFER2 (Cvel_9223)); Apicomplexa: “Tg” *Toxoplasma gondii* (TgFER1 (TGME49_309420), TgFER2 (TGME49_260470), TgFER3 (TGME49_295472 + TGME49_295468)) “Nc”, *Neospora caninum* (NcFER1 (NCLIV_053770), NcFER2 (NCLIV_026570), NcFER3 (NCLIV_002280)), “Em” *Eimeria maxima* (EmFER1 (EMWEY_00002120), EmFER2 (EMWEY_00009280), EmFER3 (EMWEY_00017650)), “Pf” *Plasmodium falciparum* (PfFER1 (PF3D7_0806300), PfFER2 (PF3D7_1455600)), “Pb” *Plasmodium berghei* (PbFER1 (PBANKA_122440), PbFER2 (PBANKA_131930)), “Cp” *Cryptosporidium parvum* (CpFER1 (cgd8_2910), CpFER2 (cgd2_2320)), “Gn” *Gregarina niphandrodes* (GnFER1 (GNI_063830), and GnFER2 (GNI_073830)). Alignment and unrooted Jukes-Cantor phylogenetic tree were generated in Geneious (v.6.1.6) [44]) from a MUSCLE alignment using neighbor-joining. Note that the FER1 and FER2 nodes for Tg and Nc are barely discernable at this scale. From [43].

The versatility of the PKC-C2 domain family is further enhanced by domains of tandemly positioned C2 domains creating a double C2 or DOC2 domain. This provides more flexibility in the interaction repertoire by creating cooperatively functioning C2 domains. Importantly this effect can modulate the response to varying [Ca^2+^]_c_. DOC2 proteins control numerous Ca^2+^-dependent exocytic processes, but have been studied in most detail for the Ca^2+^-dependent release of neurotransmitter from neurons [22,25,26,27] (Figure 2). For Ca^2+^ mediated fusion of vesicles to the plasma membrane to occur in general, a DOC2 Ca^2+^-sensor with a transmembrane (TM) domain anchored in the secretory vesicle is required, represented by the synaptotagmin (Syt) family [22,28,29,30]. These typically respond to fast, high Ca^2+^ fluxes and are required for fast-synchronous neurotransmitter release [23,31,32,33]. Soluble sensors like mammalian DOC2 proteins (Figure 2B: note that their domain structure is very different from TgDOC2) are sensitive to lower [Ca^2+^]_c_ concentration and regulate constitutive and spontaneous asynchronous neurotransmitter release [23,34,35,36,37]. Upon the correct Ca^2+^ concentration, DOC2 proteins act on congregating and clamping on SNARE proteins residing in the secretory vesicle (v-SNARE) and target membrane (t-SNARE) and result in fusion of the two membranes, thereby releasing the vesicle contents.

The ferlin family comprises a distinct family of DOC2 proteins absent from neurons that facilitate secretory processes in other cell types. Ferlins are defined by a C-terminal TM domain and 5–7 C2 domains, typically organized in three DOC2 domains (Figure 2). Vertebrate ferlins function in the secretory, endocytic and lysosomal pathways with structural and functional importance in health and disease [38]. Secretion of synaptic vesicles from the inner ear cells upon mechanical stimulation by sound, only a single Ca^2+^-sensor, otoferlin, is required for exocytosis, at the site of secretion, thereby deviating significantly from the standard model of Ca^2+^-dependent exocytosis established in neurons [39,40,41]. Humans encode six ferlins that cluster closely together compared to the three different apicomplexan ferlins (Figure 2C). This suggests that the apicomplexan diversity represents unique clades that originated independently of the mammalian ferlins, which appear to have radiated out of a single ancestral mammalian ferlin.

Here, we discuss the apicomplexan ferlins in light of newly emerging insights regarding the triggers and machinery facilitating microneme and rhoptry exocytosis. In addition. *T. gondii* ferlin and TgDOC2 mutants were used to explore their respective contributions to the various secretion events, which fortuitously led to the identification of a novel microneme protein family, MIC21, conserved in the Coccidia sub-class of the Apicomplexa.

## 2. Materials and Methods

### 2.1. Parasites and Host Cells 

Transgenic derivatives of the RH strain were maintained in human foreskin fibroblasts (HFF) or hTERT immortalized HFF cells as previously described [45]. The following previously reported parasite lines were used: line *ts*-DOC2 is the recapitulated temperature sensitive (*ts*) mutation [46] in a RHΔHX background with an addition of a C-terminal 5xTY tag on DOC2 [47], parasite line DN-FER1 is conditional overexpression of an exogenous FER1 gene controlled by the destabilization DD domain and the ligand Shield-1 with an additional Myc-tag [46], and in parasite line FER2-cKD the FER2 gene is placed under a tetracycline regulatable promoter with an additional N-terminal Myc-tag [47]. 

### 2.2. Cloning

Plasmid pmic21-MIC21-YFP(MCS)/sagCAT was cloned by PCR amplification of genomic DNA containing the ORF and the 1400 bp promoter upstream of the newly annotated start codon using primers #5085 gPro234380YFP-F GCCGGTGTTAGGAGATGACGAGACGTTTAAACCGCTGCCAGTGGATATATCTACGG and #5086 gPro234380YFP-R CTCCTCGCCCTTGCTCACCATCCTAGGAAGGTATTTCTTCAAAAGGTGTCAAGGG (restriction sites underlined) which was cloned into *Pme*I/*Avr*II digested ptub-YFP-YFP(MCS)-3′dhfr/sagCATsag [48] to replace the α-tubulin promoter and the first YFP ORF by Gibson assembly (NEB).

### 2.3. SILAC and ESA Collection

We adapted published methods to differentially label *T. gondii* cultures with isotopes [49]. Parasites grown at the permissive condition (35 °C) were metabolically labeled with heavy (C13, N15) Lys and Arg (L-Lysine-2-HCl (Thermo/Life Cat # 89987) and L-Arginine-HCl (Thermo/Life Cat # 88210;) for two lysis rounds using established *T. gondii* SILAC protocols [49]. Medium was prepared in phenol free DMEM Media for SILAC (Thermo/Life Cat # 89985). Specifically, parasites were grown in 5 mL medium (containing 1% FBS) in a T25 flask that was pre-equilibrated with isotope containing medium 24 hours (hrs) prior to parasite inoculation. Following lysis 48 hrs later, parasites were passed in one T25 flask under similar conditions. 5 mL freshly lysed parasites were passed into two T175 flasks per condition, pre-equilibrated for 24 hrs in isotope containing medium. Following growth for 48 hrs at the restrictive condition (40 °C, 1 μg/mL anhydrous tetracycline (ATc), or 1 μM Shield-1) in isotope labeled medium, or 48 hrs at the permissive condition (35 °C or no ligands) in non-isotope labeled medium, monolayers were scraped with a rubber policeman and parasites released by passage through a 21 gauge needle. Parasites were collected by centrifugation at 1000× *g* for 20 min at RT, resuspended in 1 mL of serum free SILAC medium containing 10 mM Hepes pH 7.3 and centrifuged again. Following one additional wash step, parasites were resuspended to 1.2 × 10^8^ parasites/mL in serum free SILAC medium. 200 μL aliquots were placed in round bottom 96 well plates ((CELLTREAT Scientific Products)). Secretion was triggered by 2% ethanol incubation at 37 °C for 15 min as described [50,51]. The plate was centrifuged at 1000× *g* at 4 °C for 10 min to pellet cells and 100 μL of the supernatant was transferred to an Eppendorf tube followed by addition of 10 μL protease inhibitor (100× stock; Sigma # P88490). Parasites and supernatant remaining in the plate were resuspended and transferred into a separate set of tubes, centrifuged at 1000× *g* at 4 °C for 10 min, supernatant discarded and pellet resuspended in 180 μL PBS before addition of 20 μL 10% SDS as control for parasite number. Protein concentrations were determined using the BCA plate assay (Pierce #23227).

### 2.4. Silver Staining 

Silver staining of SDS-PAGE gels was performed using a Silver Stain Kit (Pierce, Thermo Scientific) following the manufacturer’s protocol. Briefly, 25 µg each of Heavy Labelled (HL) and Light Labeled (LL) ESA proteins were run on 8–12% SDS-PAGE MOPS gel. The gel was washed twice 5 min in water and fixed in 30% ethanol/10% acetic acid for 2 × 15 min. The gel was washed twice in 10% ethanol for 10 min followed by 2 washes for 1 min in water. The gel was sensitized for 1 min in Sensitizer working solution followed by 2 × 1 min washes with water. Proteins were stained for 30 min in Stain working solution followed by 2 × 20 sec washes with water. Gels were developed in Developer Working Solution for 3 min and the reaction stopped with 5% acetic acid for 10 min. 

### 2.5. LC-MS/MS Analysis

LC–MS/MS analysis was performed on an LTQ-Orbitrap XL mass spectrometer (Thermo Fisher) coupled to an EASY-nLC 1000 nanoLC (Thermo Fisher). Samples were pressure loaded onto a 250 µm fused silica desalting column packed with 4 cm of Aqua C18 reverse-phase resin (Phenomenex). The peptides were then pushed onto a column (100 µm fused silica with a 5 µm tip, packed with 10 cm C18) and eluted with a gradient of 0–55% Buffer B in Buffer A (Buffer A: 95% water, 5% acetonitrile, 0.1% formic acid; Buffer B: 20% water, 80% acetonitrile, 0.1% formic acid). The flow rate through the column was set to 400 nl/min and the spray voltage was set to 3.5 kV. One full MS1 scan (FTMS; 400–1800 MW) was followed by seven data dependent scans (ITMS) of the n^th^ most intense ions with dynamic exclusion enabled. 

The generated tandem MS data were searched using the SEQUEST algorithm [52] using a concatenated target/decoy variant of the *T*. *gondii* GT1 ToxoDB-V28 database combined with a target/decoy non-redundant variant of the human UniProt database. Data sets were searched independently with the following parameter files; for the light search, all amino acids were left at default masses; for the heavy search, static modifications on lysine (+6.02013) and arginine (+10.00826) were specified. A static modification of +57.02146 on cysteine was specified in all searches to account for iodoacetamide alkylation. SEQUEST output files were filtered using DTASelect 2.0 [53]. Reported peptides were required to be unique to the assigned protein (cannot be attributed to both human and *T. gondii* derived protein), with a minimum of two unique peptides per protein. Discriminant analyses were performed using the CIMAGE quantification package as previously described [54]. The H/L ratios generated for unique peptides were grouped by protein with the median H/L ratio chosen as the representative ratio for that protein. Human proteins were removed from the dataset. Ratios from each sample were normalized to the median ratio of *T. gondii* derived proteins within that sample in order to correct for variations in isotope labeling. 

### 2.6. Fluorescence Microscopy

RHΔHXGPRT parasites were co-transfected with 15 μg each of plasmid pmic21-MIC21-YFP(MCS)/sagCAT and PG53 encoding MIC8-mCherry ([55] a kind gift from Markus Meissner) or pMIC2-mCherry-Myc (Luc in plasmid pMIC2-Luc-Myc, kindly provided by David Sibley [56], was replaced with mCherry). 18 hrs post transfection, parasites were imaged with a Zeiss Axiovert microscope and a ORCA-FLASH 4.0 camera was used in capturing images using 100x objective with oil and 0.55 Numerical Aperture (NA).

### 2.7. Western Blots

SDS-PAGE (8–12%, MOPS) separated ESA samples and corresponding total parasite lysates were blotted onto PVDF membrane and probed with mouse α-MIC2 (1:8000) [57], mouse α-TY (MAb BB2; 1:1000; a kind gift from Sebastian Lourido), mouse α-tubulin (12G10) (1:2000; Developmental Studies Hybridoma Bank, Iowa City, IA, USA), rabbit α-GRA7 (1:2000; a gift from John Boothroyd) [58]. Procedures and signal intensity quantification as previously described [43,47]. A two-tailed, two sample equal variance Student’s *t*-test was performed on protein levels normalized to α-tubulin. 

### 2.8. MIC21 Phylogeny

The reannotated TGGT1_234380 with signal peptide was used for a BLASTP search of select representative Apicomplexa against EuPathDB [59]. Manually curated ortho- and para-logs were aligned using ClustalO 3.0 (CLC Main Genomic Workbench 20, Qiagen). The alignment was used to generate an unrooted phylogenetic tree using the Neighbor joining Algorithm, Jukes-Cantor protein distance and 100x bootstrap analysis (CLC Main Genomic Workbench 20, Qiagen). The following sequences were included in the analysis: MIC21: TGGT1_234380, HHA_234380, NCLIV_049520, CSUI_003785; MIC21-L1: TGGT1_222188, HHA_222188, NCLIV_005580, SN3_01200570, CSUI_000781 (2a), CSUI_009221 (2b); MIC21-L2: EBH_0032420, ETH_00028875, EAH_00066010, LOC34621018; MIC21-L3: ETH_00004565, LOC34624209. 

## 3. Results

### 3.1. Apicomplexan Protein Trafficking to the Secretory Organelles

The secretory pathway that subsequently passes through the endoplasmic reticulum (ER) and Golgi apparatus mediates protein trafficking to the three secretory organelles. Targeting proteins to the dense granules does not require any additional signals and comprises the default pathway secretory organelle. Specifics on the protein trafficking pathways to the micronemes [60] and rhoptries [61] and their biogenesis were recently reviewed. Although some rhoptry specific targeting signals have been identified [62,63], specific sorting signals for microneme proteins have remained elusive. Following the Golgi compartment, microneme and rhoptry proteins traffic through an endosome like compartment (ELC) (reviewed in [64,65]), where they undergo maturation by proteolytic removal of a pro-peptide [66]. Specific Rab GTPases have been associated with the first leg of the trafficking through the endosomal pathway, but are absent from the mature organelles or their sites of secretion [67]. Peculiarly, a subset of microneme proteins (MIC3, MIC5, MIC8 and MIC11) traffic, in Rab5A/C-dependent fashion while the others are Rab5A/C-independent [67]. Sustained microneme secretion in gliding extracellular parasites is balanced with active endocytosis [68]. During cell division, micronemes of the mother are re-directed into the newly forming daughters, which hints at a mechanism that supports trafficking of micronemes in more than one direction [69].

### 3.2. Organization of Organelle Secretion: Small Molecules and Machinery

Microneme release is turned off during intracellular replication and only occurs during egress, gliding and host cell invasion (Figure 1C). Pharmacological studies allowed for dissection of the controls of microneme exocytosis and established a role for three signaling molecules: cytoplasmic Ca^2+^, phosphatidic acid (PA) and cGMP [70]. The levels of cytoplasmic calcium ([Ca^2+^]_c_) correlate with the level of microneme secretion [71] (Figure 1C), and the micronemes are considered a *bona fide* Ca^2+^-dependent secretory organelle [72]. Ca^2+^ mediators are involved at several levels. Firstly, the exocytosis event has been associated with Centrin2 [73], which has four EF-hand domains putatively binding Ca^2+^, and two double C2 domain (DOC2) proteins, TgDOC2 [46] and FER1 [47]. An indirect role is also found in the requirement of a Ca^2+^-dependent protein kinase, CDPK1, for microneme exocytosis, but the key phosphorylation events are still unknown [74,75]. A parallel set of controls is mediated by phosphatidic acid (PA) produced upon activation of egress which is deposited in the plasma membrane. PA is recognized by an essential acylated pleckstrin homology (PH) domain-containing protein (APH) on the surface of the micronemes [76]. Micronemes are exocytosed at the very apical tip of the parasite, where the rhoptries are discharged as well.

The triggers for rhoptry exocytosis are not well understood besides that this requires microneme secretion first, and that it only occurs upon contact with a host cell. The connection to the micronemes resides in critical roles for microneme proteins MIC8 [55], AMA1 [77] and CLAMP [78], but the detailed mechanisms have not been resolved. In addition, rhoptry secretion is mediated by PA and phosphatidylinositol 4,5 bisphosphate (PIP2) [79], which accumulate at the plasma membrane following induction of the microneme secretion [77]. C2 and PH domains in Rhoptry Associated Surface Proteins (RASPs) engage with PA and PIP2, bringing the rhoptry in close contact with the plasma membrane and/or apical vesicle residing at the very apical between the rhoptry and the plasma membrane. [70]. In addition, a machinery shared with the release of trichocysts by ciliates comprises several Nd proteins, names for their ‘not discharged’ genetic phenotype in the ciliate *Paramecium* [80]. The Nd proteins are critical for the apical rosette, a structure in the plasma membrane through which the rhoptries and trichocysts are secreted. The apical vesicle is positioned on the cytoplasmic side of the rosette opposed to the rhoptry tip. It is of note that microneme secretion is not mediated by the rosette as they are positioned at the plasma membrane next to the apical vesicle and, moreover, microneme secretion is also not affected by disruption of the rosette structure in TgNdP1 and TgNdP2 mutants [80]. Some Nd proteins reside in the cytoplasm and others at the apical vesicle, but the Nd proteins are either directly critical for the rosette structure, or for the exocytosis event [80]. Furthermore, the Nd proteins interact with another DOC2 domain protein, FER2, which is essential for rhoptry secretion [43]. Two models have been proposed for the role of Nd proteins in controlling the fusion step: 1. the correct assembly of Nd6, Nd9, NdP1 and NdP2 as a complex together with a GTPase (TGME49_277840) is mediated by the Nd6 guanine exchange factor (GEF) domain and Ca^2+^-dependent action of FER2, or; 2. cytosolic Nd9 and/or NdP1 interact transiently with the apical vesicle associated Nd6 to trigger the assembly of the rosette [80]. Although no pharmacological compounds are known to trigger rhoptry secretion, an inhibitor, 4-bromophenacyl bromide (4-BPB), is available [81]. This compound typically inhibits A2 phospholipases, but its target in *T. gondii* is unknown [81]. Overall, the structural architecture of the site of rhoptry secretion comprises a critical role for several C2 domain proteins engaging with various lipids, while providing a putative role for Ca^2+^ through FER2 [79].

Dense granule secretion is the least understood. Not even the site of secretion has been conclusively established. Although there is evidence that the dense granules are secreted somewhere at the apical end of the parasite [82,83], their secretion has also been inferred from a specialized invagination at the posterior end of the parasite where the basal complex resides [84]. However, an alternative interpretation is that dense granule proteins are secreted at an apical site from the parasite and subsequently transition to the posterior end [85]. In either scenario dense granule proteins facilitate the assembly of multilamellar vesicles in the vacuole at the posterior end of the parasite, which subsequently transition into tubulated structures making up an intravacuolar tubulovesicular network [84]. Since dense granules with different contents are present, it is also possible these might have distinct secretion sites and/or kinetics. 

### 3.3. Ferlins in Mammals

The mammalian ferlins come in two basic flavors differentiated by their sub-cellular localization at either the plasma membrane or on intracellular compartments. Localization relates to their function in either late endosomal transit or trans-Golgi recycling [86]. Dysferlin and otoferlin have been the most studied. Dysferlin is critical for muscle development and functions in vesicular trafficking and lysosome exocytosis during muscle plasma membrane damage repair [38,87]. Dysferlin does not localize to lysosomes in intact myotubes, but dysferlin-containing vesicles fuse with lysosomes upon sarcolemma damage, possibly in response to a raise in [Ca^2+^]_c_ [88]. This two-step process might mimic the role of the apical vesicle wedged between the rhoptry tip and the plasma membrane, which also required a two-step fusion event [80]. A further parallel with the Apicomplexa is the indication that rhoptry targeting tyrosine motifs are shared with mammalian lysosomal trafficking signals [63,89]. This suggests a kinship at several levels between mammalian lysosomes and rhoptries, with a possibly comparable role for dysferlin and FER2, respectively. 

Otoferlin functions as both a scaffolding protein in the secretory pathway as well as in the actual membrane fusion during exocytosis [39,41,90,91]. Otoferlin is expressed in many tissues but in Cochlear Hair Cells (CHCs) in the inner ear auditory systems where is localizes to synaptic vesicles and the plasma membrane and is essential in neurotransmitter release upon mechanical triggers: a variety of mutations across otoferlin lead to hearing loss [90,92]. Under low [Ca^2+^]_c_ interactions among C2C, C2D, C2E, and C2F is promoted, whereas high [Ca^2+^]_c_ leads to interaction with phospholipids, the prelude to membrane fusion [90].

### 3.4. DOC2 Protein Repertoire in T. gondii

The Apicomplexa encode two widely conserved ferlin proteins, FER1 and FER2 ([43] and one quite unconventional alveolate-wide conserved DOC2 protein without a TM domain [46] (Figure 2A,C). Some parasites, including *T. gondii*, encode a degenerate third ferlin, TgFER3 [43]. We were able to knock out TgFER3 without any effect on the lytic cycle and concluded that it is not involved in organelle secretion, at least not in the tachyzoite stage (Tagoe and Gubbels, unpublished). TgFER1 and TgFER2 mediate exocytosis of the micronemes and rhoptries, respectively [43,47]. Besides *T. gondii*, ferlins have only been studied in one other apicomplexan parasite, *Plasmodium berghei*. The ferlin-like protein, PbFLP, the TgFER1 ortholog, is essential for male gametocyte egress, and its knock out phenotype was consistent with a role in exocytosis [93]. 

The conserved DOC2 protein without TM domain, TgDOC2, is unconventional in its relatively long length without other recognizable domains (Figure 2). TgDOC2 is essential for microneme protein secretion, a function that is conserved in *Plasmodium falciparum* [46]. PfDOC2 has been shown to bind Ca^2+^ and is present in the membrane fraction of replicating parasites [94]. In conclusion, all conserved DOC2 domain proteins function in the exocytosis of the specialized apicomplexan secretory organelles defining the obligate intracellular lifestyle of these parasites. Comparing the function and behavior of mammalian ferlins to the *T. gondii* repertoire points at otoferlin as relevant to understand FER1 and dysferlin as a role model for FER2.

Regarding the function of TgDOC2, comparison with the mammalian repertoire points to the DOC2 family: cytoplasmic Ca^2+^ sensors like mammalian DOC2 proteins (Figure 2B: note that their domain structure is very different from TgDOC2) which are sensitive to lower [Ca^2+^]_c_ concentration and regulate constitutive and spontaneous, asynchronous neurotransmitter release [34,35,36,37]. They act in concert with a membrane bound, TM domain containing Ca^2+^ sensor like the synaptotagmins or otoferlin that typically respond to fast, high Ca^2+^ fluxes required for fast-synchronous neurotransmitter release [23,31,32,33]. Tentatively, this dual player DOC2 system seems to fit the micronemes with their dynamic secretory nature (Figure 1C), whereas as single DOC2 protein seems sufficient for the single burst in rhoptry release. However, based on the unconventional structure of TgDOC2 we cannot exclude other potential roles fulfilled by different mammalian multiple C2 domain proteins such as MUNC13 or RIMs outlined in Figure 2B. Munc13 (mammalian ortholog of *C. elegans* uncoordinated protein) stabilizes the SNARE and Ca^2+^-sensor complex and is absolutely essential for any type of Ca^2+^-mediated exocytosis [95,96]. MUNC13 is not a Ca^2+^ sensor but a CAPS (Calcium-dependent Activator Protein for Secretion), which prime vesicles for Ca^2+^-triggered exocytosis. MUNC13s harbor 2 or 3 C2 domains and a diacylglycerol-binding C1 domain [97] (Figure 2B). The MUN domain stabilizes the SNARE complex [97,98]. However, the *T. gondii* genome does not encode a protein with a domain reminiscent of a MUN domain, which indicates a mechanism distinct from the well-studied systems. RIMs are involved in vesicle priming, docking and organization at the site of secretion. RIM stands for Rab3 Interacting Molecules. They harbor multiple C2 domains (Figure 2B) and bind multiple presynaptic proteins, e.g., SNAREs, Rab3, Munc13’s, mediated by the C2A, C2B and Pro-rich domains [99]. RIMs receive the secretory vesicle from the Rab-mediated trafficking machinery and relay it to the fusion proteins. Knock-out of RIMs reduces neurotransmitter release to 60% [100]. The relevance to *T. gondii* goes off the rails by the absence of Rabs and SNAREs at the sites of microneme and rhoptry secretion [67]. However, the unconventional nature might bypass the need for these players, as ferlins equally have the capacity to function without. Moreover, otoferlin is even considered to be the one and only Ca^2+^-sensor for secretory vesicle exocytosis in CHCs, since stimulus–secretion coupling is eliminated in otoferlin knock-out mice [39,40,41].

In conclusion, the role of apicomplexan ferlins in microneme and rhoptry exocytosis is consistent with mammalian ferlins acting on the endosomal pathway, wherein the micronemes and rhoptries reside. However, given the specialized nature of the apicomplexan secretory organelles combined with the ancient divergence between FER1, FER2 and the mammalian ferlin families (Figure 2C), this comparison is only at a high level. The ferlins act in the context of lineage specific proteins like TgDOC2 [46] and APH [101] for the micronemes, and RASP [79] and Nd [80] proteins in rhoptry exocytosis.

### 3.5. FER1 and FER2 in Microneme and Rhoptry Fusion with the Plasma Membrane

The fine details on how FER1, FER2 and TgDOC2 orchestrate the exocytosis of micronemes and rhoptries is associated with several questions. In particular, *T. gondii* Rabs are absent from the microneme and rhoptry secretion site in the apical conoid [67]. Although Rab11A has been shown to be critical in constitutive secretion of dense granules, this is not a triggered event [102]. In addition, no *Toxoplasma*’s SNARE proteins have been detected at the site of microneme and rhoptry secretion site in the very apical plasma membrane [103]. The most forward localizing SNARE in the secretory pathway identified is a syntaxin, TgStx12, which is critical in trafficking of proteins from ELC to the micronemes and rhoptries, but not for their secretion. Thus, this is different from neurotransmitter release where SNAREs play a key role in the membrane fusion event facilitating exocytosis.

In general, the absolute requirement for SNAREs in exocytosis, certainly regarding ferlin-mediated exocytosis, has come under debate. It has been postulated that fast, Ca^2+^-dependent exocytosis is inconsistent with the role of SNAREs [104] and some exocytosis in absence of SNAREs is possible [21,105]. Furthermore, some neurotransmitter is still released when all relevant SNAREs are depleted [105], and alternative models for SNARE independent neurotransmitter release have been postulated [104]. Most germane to the situation in parasites is the absence of SNAREs from the site of otoferlin-mediated neurotransmitter release in the auditory hair cells [106]. Furthermore, at the sites of membrane lesions in cultured myotubes SNAP23, syntaxin4 and VAMP4 do not accumulate, but myoferlin does and is required for membrane repair [107,108]. Thus, these ferlins are essential for specialized membrane fusion events, but are not dependent on SNARE proteins. This leaves the door open for SNARE-independent fusion, or alternatively, an as yet uncharacterized class of SNAREs [90,106]. 

Regarding the modular nature of the DOC2 domains in ferlins, the multiple C2 domains have been proposed to be able to support membrane binding integrating the Ca^2+^-sensing and membrane fusion events [31,42]. Indeed, pairs of C2 domain can interact cooperatively to take on a closed or open conformation [109] or coordinate a driver-facilitator relationship [110,111]. In this manner C2-C2 interactions facilitate protein scaffolding, determine Ca^2+^ sensitivity and direct lipid binding affinity. In this division of labor, a single C2 domain may bind a recruited protein, while others within the ferlin are responsible for binding Ca^2+^ or lipid. In this way all domains of the protein coordinate to allow fusion of membranes [112,113]. The combination of several C2 domains in a single protein allows for dynamic conformational changes that reveal or hide different C2 domains and thus change its function and as such ferlins might facilitate *Toxoplasma* exocytosis in absence of SNAREs. Our working model is therefore that TgDOC2 and FER1 and FER2 can fulfill the missing function of SNAREs and Rabs at the plasma membrane. 

### 3.6. Ferlins in Organellar Trafficking 

FER1 does not only function in microneme exocytosis but also in microneme organelle trafficking [47]. Two different phenotypes in different mutants were observed: conditional overexpression of dominant negative FER1 allele without its TM domain results in a reversible, retrograde microneme transport away from the apical cortex to a pile up of micronemes in an apical cytoplasmic location, whereas conditional overexpression of the full-length FER1 resulted in an anterograde transport of the micronemes into the very apical tip of the parasite and even premature exocytosis [47]. The retrograde transport was interpreted as being germane to the process of microneme recycling from the mother into the emerging daughters during the second half of cell division [69]. The actin-dependent trafficking also provides a tentative connection between FER1 and a myosin. The anterograde trafficking supports a role in replenishment of secretory vesicles at the site of secretion. It is assumed that micronemes travel along the sub-pellicular microtubules as they follow this organization pattern in their cortical localization pattern [67]. However, there is no direct experimental data supporting this assessment. The microtubule based motor, dynein TgDLC8a, has been associated with replenishment, but since it is only observed in the very apical tip, is unlikely to transport the organelles along the sub-pellicular microtubules, but more likely through the conoid [73]. Either way, dual roles in membrane fusion and vesicle trafficking are also seen in the vertebrate ferlins [86], such as otoferlin in replenishment of synaptic vesicles [40,41] through myosin VI [114,115]. The role of dysferlin in vesicular trafficking in quite diverse [38], but is also dependent on actin myosin for plasma membrane repair [116]. Overall, these observations and similarities with vertebrate ferlins indicate that the role of FER1 changes under different conditions organellar trafficking during intracellular replication, and microneme exocytosis upon activation of egress, during gliding and host cell invasion. Currently, we have no indication that FER2 has such a dual role. The physical feature of the rhoptries are in alignment with this model: they are constitutively docked at the apical end and do not traffic [80] whereas their relatively large size would also not be very amendable to an organelle recycling pathway [61]. 

### 3.7. T. gondii DOC2 Proteins Act Differentially on the Various Secretory Organelles

We performed knock-out or dominant negative allele studies on FER1 [47], FER2 [6], and TgDOC2 [46] to reveal the organelles these factors are acting on. However, to obtain more subtle, quantitative data on the exact nature of secretion events and to refine the mechanistic models we performed Stable Isotope Labeling with Amino acids in Cell culture (SILAC) proteomics on the proteins released in our set of secretion mutants. We induced all conditional mutant phenotypes overnight followed by mechanical release of the parasites from their host cells and then stimulated exocytosis with 2% ethanol for 15 min. We collected the excreted and secreted antigens (ESA) under both induced and non-induced control conditions, which were differentially labelled with light- and heavy-isotope containing amino acids, respectively, before mixing in equal ratios and analysis by mass spectrometry (Figure S1). Changes in the heavy:light ratio reveal which proteins are differentially secreted in each mutant. 

**Figure 3:**
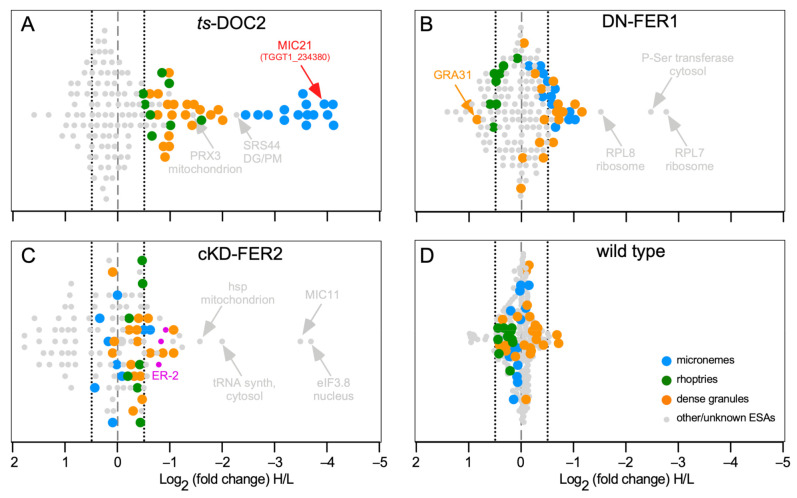
SILAC data for *T. gondii* DOC2 and Ferlin mutants. (**A**). *ts*-DOC2 is a recreated temperature sensitive allele of DOC2 conditionally treated at 35 °C and 40 °C [43,47]; (**B**). DN-FER1 is a DD-Myc conditional overexpression allele induced with 1 μM Shield-1 [47]; (**C**). cKD-FER2 is tetracycline regulated promoter replacement induced with 1 μM ATc [59]; (**D**). the wild type control consisted of RHΔKu80 parasites treated at 35 °C and 40 °C. All inductions were performed for 48 hrs on intracellularly replicating parasites. For ESA collection, parasites were mechanically lysed from the host cell, and stimulated with 2% ethanol for 15 min. Parasites grown under permissive conditions were grown in light-isotope (L) labeled amino acids; parasites at restrictive conditions in heavy-isotope (H) labeled amino acids. Equal amounts of ESA collected under permissive and restrictive conditions were mixed and subjected to mass spectrometry. Averages of two biological repeats are shown, except for cKD-FER2 which was a single experiment. Marked in pink, the cKD-FER2 experiment is the only one in which some ER proteins (ER-2 pool defined in [117]) were detected (TGGT1_229480: putative calcium binding protein precursor; TGGT1_221210: cyclophilin; TGGT1_211680; protein disulfide isomerase). Dotted lines mark the −0.5 and +0.5 Log2 arbitrary cut-offs for decreased or increased significant changes, respectively, in excretion/secretion above background.

For the temperature sensitive (*ts*)-TgDOC2 lines, the results confirmed the dominant role of TgDOC2 on micronemes (Figure 3A). We also confirmed that the *ts* allele results in protein instability and loss of TgDOC2 from the parasites at restrictive, high temperature (Figure S2). However, we also identified significantly less secretion of both the dense granules, and, to a lesser but still significant extent, the rhoptries. Induction of DN-FER1, which redirects the vast majority of micronemes away from the cortex thereby inhibiting their secretion, results in a significant reduction in secretion of many microneme proteins, whereas the ones below the significance cut-off trend strongly to reduced secretion (Figure 3B). There is no effect on the rhoptries. However, we observed a secretion trend for the dense granules, mimicking the micronemes, albeit largely below the statical relevance limit. 

### 3.8. Do DOC2 Proteins Act on Multiple Secretory Organelles?

The suggestive role of TgDOC2 in microneme and rhoptry discharge and that of FER1 in dense granule exocytosis can be interpreted in two different ways: 1. a direct role for TgDOC2 in rhoptry and dense granule exocytosis and for FER1 in dense granule exocytosis; 2. induced dense granule and rhoptry release requires secretion of the micronemes first. Discharge of rhoptries indeed requires microneme secretion first [54], which would explain the strong effect seen in the *ts*-DOC2 line. However, the dependence of dense granules on either TgDOC2 or FER1 is less clear. In fact, an opposite correlation between induced microneme and dense granule exocytosis has been reported [118]. However, dense granule exocytosis was not completely inhibited, and the differences in protein secretion we detect are of course relative to the non-induced condition. The secretion drop we see therefore drops even below the reported reduced level [118].

**Figure 4:**
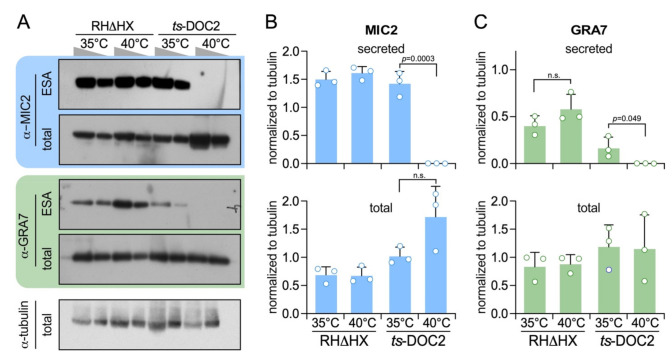
Dense granule secretion is TgDOC2 dependent. (**A**). Representative Western blots of *ts*-DOC2 secretion assays as performed for the ESA-SILAC experiment. “total” represents the pellet of the ESA assay. Antiserum against α-tubulin was used as loading control. Relative to the first lane for each condition, only half the protein amount was loaded in the second lane. (**B**). Quantification of secreted and total MIC2 normalized against α-tubulin shows that MIC2 expression is 3-fold upregulated in *ts*-DOC2 at the restrictive condition (40 °C), yet none is secreted. (**C**). Quantification of secreted and total GRA7 normalized against α-tubulin shows that GRA7 is not upregulated, but its stimulated secretion is dependent on DOC2. All blots used for quantification are provided in Supplementary Figure S2 and the quantification data in Supplementary Table S2. Statistics was performed with a two-tailed, two sample equal variance Student’s *t*-test; n.s.: non significant.

To validate the reduction in dense granule exocytosis we performed Western blots and quantified the differential secretion of the microneme and dense granules protein reporters MIC2 and GRA7, respectively (Figure 4). This revealed that like MIC2, GRA7 secretion is completely inhibited. The other notable new insight is that the expression levels of both MIC2 and GRA7 are trending upward upon TgDOC2 depletion. Although this upregulation does not reach a statistically significant difference, it trends contrary to the complete disruption of secretion, thereby indicating the potential presence of a feedback loop to increase protein expression as a compensatory mechanism for the block in exocytosis.

### 3.9. Identification of a Novel Microneme Protein: MIC21

**Figure 5:**
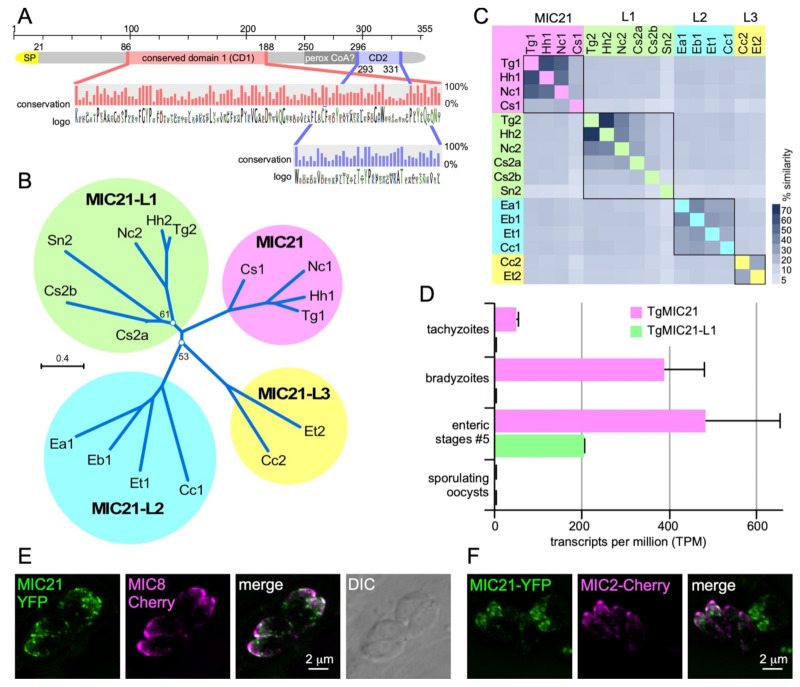
Characterization of putative microneme protein TGGT1_234380. TGGT1_234380 was the top hit in the *ts*-DOC2 SILAC experiment and annotated as a hypothetical protein on ToxoDB. Recently, the protein was detected in the microneme fraction by HyperLOPIT [117]. Querying select, representative apicomplexan and chromerid annotated genomes in EuPathDB identified only orthologs in the Coccidia. All except the *T. gondii* annotation of the predicted gene contained an N-terminal signal peptide (SP), which upon closer examination, was also present in the *T. gondii* gene if the gene was not spliced. This re-annotated version of the protein, which we named microneme protein 21 as this is the next available number in the microneme protein family (MIC21), was used to assemble a protein alignment that is the basis of data presented in panels A-C. (**A**). Schematic representation of MIC21. Two highly conserved domains (CD1 and CD2) were identified whose conservation across orthologs and paralogs is shown in the magnified panel as % conservation and amino acid logo plots. A PFam search identified domain defined in the peroxisomal acyl-CoA oxidase-II domains 3 and 4 (SCOP domain d1is2a3) with a non-significant e-value of 1, and furthermore this domain is not conserved across ortho- and para-logs. (**B**). Unrooted (neighbor-joining, Jukes-Cantor, CLC Workbench) phylogenetic tree of representative MIC21 related sequences identified in the Coccidia resolves into 4 branches of ortho- and paralogs (MIC21 cluster next to MIC21-like proteins MIC21-L1, MIC21-L2, and MIC21-L3 clusters). Bootstrap analysis (100x) supported all branches with over 70%, except for the two branch points as indicated. Hh: *Hammondia hammondi*; Nc: *Neospora caninum*; Cs: *Cystoisospora suis*; Sn: *Sarcocystis neurona*; Ea, Eb, Et: *Eimeria acervulina*, *brunetti* and *tenella*, respectively; Cc: *Cyclospora cayetanensis*. (**C**). Heatmap representing the percentage of sequence similarity across the DSP1 protein family. Percentage similarity based on ClustalOmega alignment. (**D**). RNAseq data available on ToxoDB reveals MIC21-L1 is not expressed in tachyzoite but only in the enteric stages. MIC21 is robustly expressed tachyzoites, bradyzoites and the enteric stages [120]. Neither MIC21 nor MIC21-L1 is expressed in sporulating oocysts [121]. Error bars represent standard error [120]. (**E**,**F**). Exogenous, transient expression of genomic DNA spanning the TgMIC21 promoter (1.4 kb) and coding sequence N-terminally fused to the YFP reporter co-transfected with a MIC8-mCherry (**E**) or MIC2-mCherry (**F**) reporter indicates MIC21 resided in the micronemes though does not perfectly co-localize with either MIC8 or MIC2.

Besides the annotated microneme, rhoptry and dense granule proteins, we also identified differentially secreted proteins outside these categories. These were mostly typical contaminants such as SAG surface antigen, ribosomal proteins, as well as mitochondrial or cytoplasmic proteins (although we cannot exclude the possibility that their secretion is truly dependent on the DOC2 domain proteins; Figure 5 and Table S1). Either way, one protein in the *ts*-DOC2 dataset stood out for its extremely strong differential secretion putting it right between the other microneme proteins: TGGT1_234380 (named MIC21; Figure 3A). This protein lacked a signal peptide prediction in ToxoDB [119], but was identified in the microneme fraction of the recent subcellular compartment proteome hyperLOPIT study of *T. gondii* [117]. Furthermore, we detected orthologs and a paralog across the Coccidia, which in contrast to TGGT1_234380 all contained a signal peptide. We therefore scrutinized the TGGT1_234380 annotation and noted a continuous open reading frame omitting the single intron that results in inclusion of a signal peptide comparable to the para- and orthologs (Figure 5A). We further identified two conserved domains (CD1 and CD2), without significant homology to known domains, which therefore does not direct further functional roles. 

Phylogenic analysis revealed that the ortho- and paralogs formed four subgroups, two of which span the cyst-forming coccidia (*Hammondia hammondi, Neospora caninum, Sarcocystis neurona, Cystoisospora suis*) whereas the other two are present in the non-cyst forming coccidia (*Eimeria* spp. and *Cyclospora cayetanensis*) (Figure 5B,C). Analysis of *T. gondii* stage specific gene expression data revealed that TGGT1_234380 is expressed in tachyzoites, bradyzoites and merozoites but not sporozoites, whereas the paralog TGGT1_222188 (MIC21-L1) is restricted to the merozoites (Figure 5D). Finally, we experimentally validated localization of MIC21 to the micronemes by exogenous expression of a C-terminal YFP fusion construct under control of the endogenous MIC21 promoter. MIC21 localized to the apical region reminiscent of the micronemes. Co-transfections with either MIC2-mCherry or MIC8-mCherry reporters showed partial co-localization, whereas MIC21 always was present in larger apical puncta (Figure 5E,F). These puncta likely reflect some mis-trafficking induced by the fusion protein, which fits with the inability to obtain parasites stably expressing the MIC21-YFP protein. Altogether, we conclude that TGGT1_234380 is a *bona fide* microneme protein and we named it MIC21; the as yet unvalidated paralogs were named MIC21-like proteins MIC21-L1, MIC21-L2, and MIC21-L3.

## 4. Discussion

Pharmacological triggering of microneme release facilitated studies on the proteins discharged by the parasite from this organelle [50,51]. Our quantitative proteomics using the DOC2 and ferlin secretion mutants confirmed the previously assigned roles for TgDOC2 and FER1 in microneme exocytosis, but in addition revealed cross-organelle impacts on the discharge dynamics of the various organelles. Notably, TgDOC2 depletion also resulted in decreased rhoptry and dense granule protein levels in the ESA (Figure 3A and Figure 4). The former might not be surprising given that microneme exocytosis is a requirement for rhoptry expulsion, but the connection to reduced dense granules release was not immediately obvious. This hints at a potential role for TgDOC2 as a general factor in secretory organelle fusion with the plasma membrane. We envision a dual function for TgDOC2, with a Ca^2+^-dependent role on microneme membrane fusion with the plasma membrane, whereas it might be a general factor, less dependent on Ca^2+^, in the membrane fusion event of rhoptries and dense granules, e.g., as a MUNC13-like clamp to stabilize the complex of molecules required for membrane fusion. 

The dynamic of the overexpression of a dominant negative FER1 allele indicated this was acting equally on micronemes and dense granules, but not on the rhoptries at all. The microneme phenotype in the DN-FER1 line originates in mistrafficking of micronemes, while the presence of a wild type FER1 allele still facilitates a low level of microneme protein secretion [47]. We did not assess dense granule trafficking in the DN-FER1 line and therefore we can currently not differentiate a role for FER1 in dense granule trafficking effect from a function in actual dense granule membrane fusion with the plasma membrane. A putative model should consider the two modes of dense granule exocytosis: a steady, constitutive level of dense granule release, and a specifically regulated burst upon completion of host cell invasion (Figure 1). It is possible that FER1 plays a differential role in these different events. 

TgDOC2 and the ferlins are derived from the ancestral PKC family of C2 domains that binds Ca^2+^. However, not all extant C2 domains retained the Ca^2+^-binding capacity, and this is an unsettled question for the apicomplexan family members. We analyzed the canonical C2 domains and determined that at least one C2 domain has a minimal set of negatively charged aspartic acid or glutamic acid residues in the key position in the C2 domain loops (FER1-C2D, FER2-C2F and TgDOC2-C2B: Supplementary Figure S3). Overexpression of FER1 allele with a C2D domain wherein the Ca^2+^-binding residues were mutated resulted in distinct microneme trafficking phenotype, suggesting physiological relevance of this in sillico analysis [47]. However, these assignments need experimental validation. It is of note that dysferlin and otoferlin each only have three predicted Ca^2+^-binding sites, but experimentally, all seven dysferlin C2 domains and five of the six otoferlin C2 domains were able to bind Ca^2+^, albeit, to variable degrees [122,123]. Hence, the presented prediction could underestimate the Ca^2+^-biding capacity in the *T. gondii* proteins.

Details on lipid and membrane binding capacities are another open question key to understand the role of the ferlins and TgDOC2 in membrane fusion. In particular the possibility to ‘sculpt’ the membrane structure of phosphatidylserine membranes as a prelude to membrane fusion [124] is of interest for FER1 since this is a membrane lipid preferentially flipped by the guanyl-cyclase-flippase required for microneme discharge [125]. Furthermore, PIP2 and PIP4 are common lipids bound by ferlins [123,126], which again are lipids with critical roles in apicomplexan exocytosis [127]. Furthermore, (conditional) lipid binding of the key RASP and Nd proteins associated with rhoptry release suggest that lipid binding by FER2 is quite likely an essential event in this process [79,80]. Furthermore, rhoptry secretion requires recruitment of all Nd proteins to the apical vesicle or rosette, suggestion protein-protein interactions are another critical aspect wherein FER2 could participate. Thus, the spatiotemporal conditions that could change ferlin conformation range from the local availability of lipids in the plasma membrane (e.g., phosphatidylserine [125] and phosphatidic acid [101] for the micronemes) to the increase in [Ca^2+^]_c_ as well the potential contribution of Ca^2+^-dependent phosphorylation by, e.g., CDPK1 [74], PKA [128] or PKG [129,130]. For example, phosphorylation of otoferlin regulates the Ca^2+^-sensitivity of C2C and C2F domains [131]. Currently there is no data supporting phosphorylation of FER1 or FER2, but three phosphorylated residues of TgDOC2 are contained in the tachyzoite phosphoproteome [132]. Furthermore, a vacuolar H^+^-ATPase (VP1) in a compartment at the very apical tip of the parasite has been related to a critical role in microneme secretion [133,134]. The identity of this compartment is not known, but it is unlikely the apical vesicle that is not essential for microneme secretion. 

In summary, the DOC2 proteins of *T. gondii* are woven deeply throughout the fabric of the exocytic organelles and events. The endosomal ancestry of these organelles fits well with endolysosomal context wherein the vertebrate ferlins facilitate vesicular trafficking and Ca^2+^-dependent exocytosis. Open questions regarding the mechanism of how the DOC2 proteins exert their function pertain to their Ca^2+^, lipid and protein binding capacities together with conditional conformational changes that might integrate environmental conditions and post-translational modifications. 

## Data Availability

The mass spectrometry proteomics data have been deposited to the ProteomeXchange Consortium via the PRIDE [135] partner repository with the dataset identifier PXD024210.

## Supplementary Materials

The following are available online at https://www.mdpi.com/2075-1729/11/3/217/s1, Figure S1: Silver stained SDS-PAGE gels of ESAs, Figure S2: Western blots of ESAs, Figure S3: In sillico analysis of Ca^2+^-binding potential of C2 domains, Table S1: ESA mass spectrometry SILAC data, Table S2: Western blot quantification data.
