# Supplementary material for: Ferlins and TgDOC2 in Toxoplasma Microneme, Rhoptry and Dense Granule Secretion
Source: Life (Basel). 2021 Mar 9;11(3):217. doi: 10.3390/life11030217 (PMC7999867; doi:10.3390/life11030217)
Supplement: Supplementary file 1 [file life-11-00217-s001.zip › supplementary.v1-3.8/Supplemantary.docx]

Supplementary Material

Ferlins and TgDOC2 in *Toxoplasma* Microneme, Rhoptry and Dense Granule Secretion


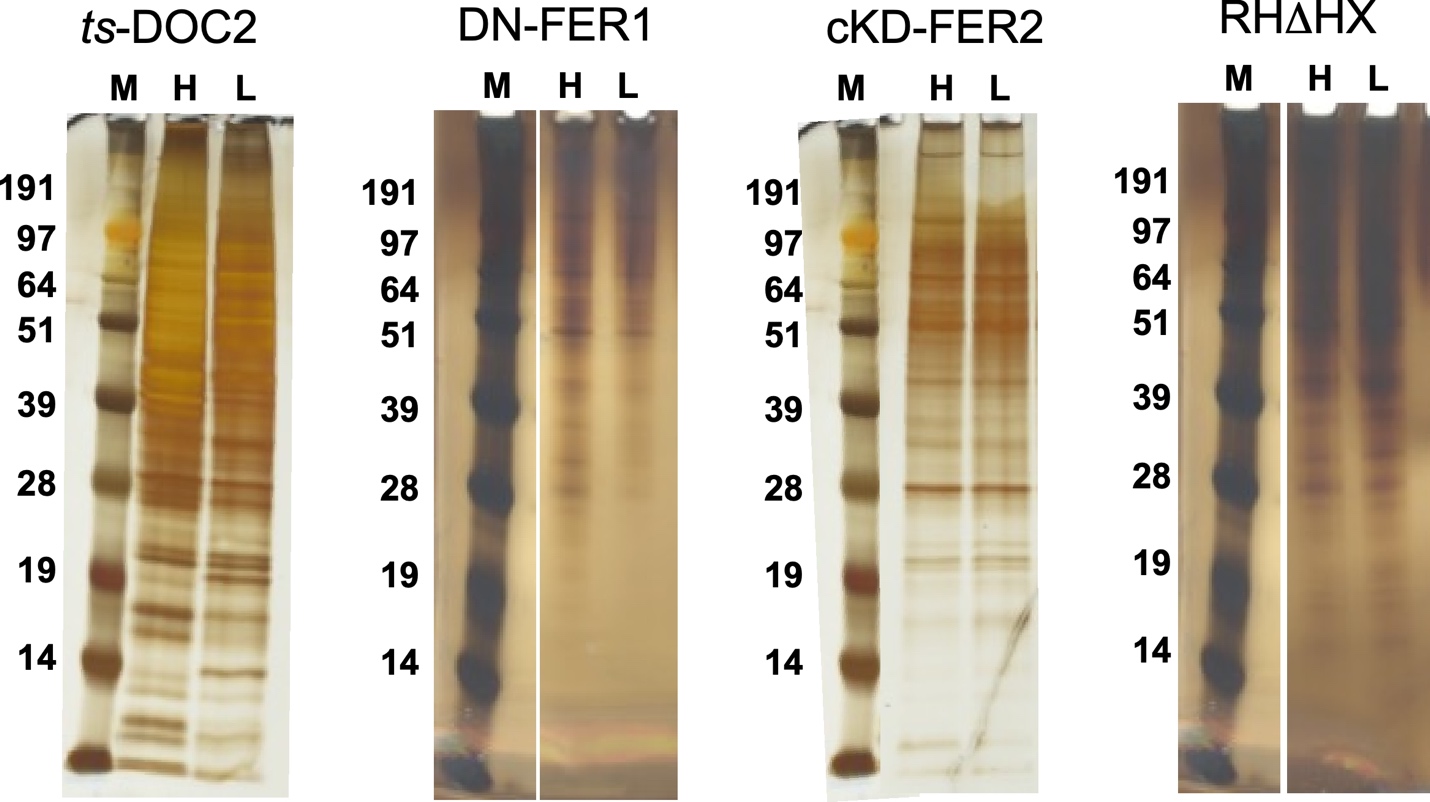


**Figure S1.** Silver stained SDS-PAGE gels of ESAs. Silver-stained gels of *ts*-DOC2, DN-FER1, cKD-FER2 and RHΔKu80 *T. gondii* lines under permissive (H: heavy amino acid labeled) and restrictive (L: light amino acid labeled) conditions. 50 μg ESA protein samples to compare relative protein abundance under each condition. Samples for each respective condition were mixed in a ratio of 1:1 and then analyzed by mass spectrometry. *ts-*DOC2 and RHΔKu80 at 35°C (H) and 40°C (L); DN-FER1 without (H) and with Shld1 (L); cKD-FER2 with (H) and without ATc (L). The lane marker M represents the SeeBlue plus 2 molecular weight ladder.


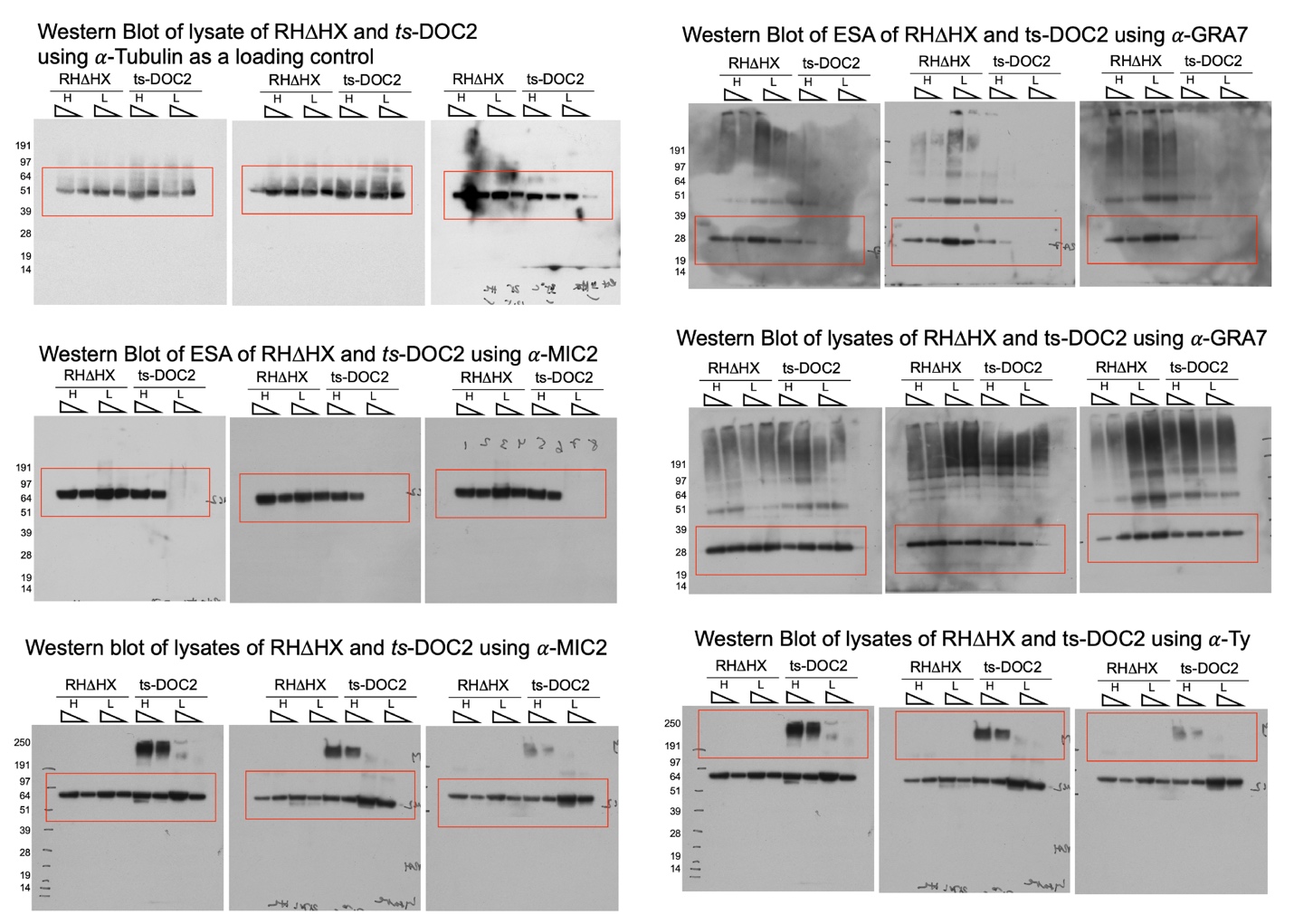


**Figure S2.** Western blots of ESAs. Triplicate western blots of three biological replicates of the ESA assay including the corresponding total lysate samples for the *ts*-DOC2 and RHΔKu80 parasite lines using mouse α-MIC2, mouse α-tubulin, rabbit α-GRA7, and mouse α-Ty, where indicated. Two lanes per sample were loaded (30 and 15 μg) were loaded for each set of western blots. After mouse α-MIC2 probing, blots were stripped for rabbit α-GRA7 probing. The red-boxed area marks the signals that were quantified (see Supplementary Table S2).


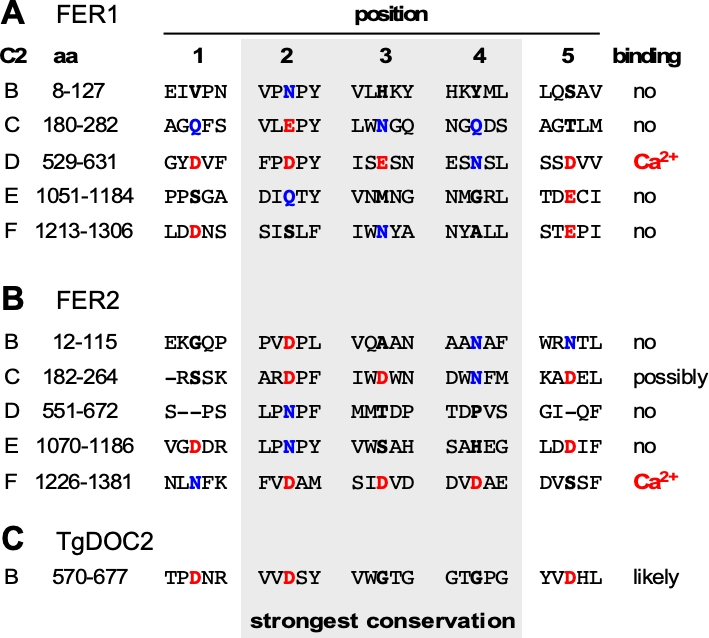


**Figure S3.** *In sillico* analysis of Ca2+-binding potential of C2 domains. FER1 (A), FER2 (B), and TgDOC2 (C) sequence analysis of the conserved key positions (#1-5) in the C2A-F domain loops interfacing with Ca^2+^ [1]. The De domain in the ferlins, and the C2A domain in TgDOC2 are too different from the consensus C2 motifs to accurately predict binding capacity. D or E residues (red) stabilize Ca^2+^, N or Q (blue) are expected to support phospholipid binding. Positions 2, 3, and 4 shaded in grey are more strongly conserving functionality than positions 1 and 5.

**Table S1.** ESA mass spectrometry SILAC data. This spreadsheet shows the various samples and their peptides identified from MS. Each condition was normalized to its respective Heavy/Light (H/L) median ratio. Mass spectrometry was performed on two biological replicates (except cKD-FER2) made up of two technical replicates for each biological sample. Proteins assignment to the various organelles were manually curated, insights from hyperLOPIT localization assignments [2], mapping of GRA proteins by BioID [3], and various other reports on the localization of specific microneme, rhoptry and dense granule proteins most of which are present in the “user comments” section on ToxoDB (Release 49 beta, 5 Nov 2020).

**Table S2.** Western blot quantification data. Quantification of Relative Fluorescence Unit (RFUs) from triplicate western blots in supplementary figure S3. Relative Fluorescence Unit (RFUs) for each band of each sample were averaged across the 15 and 30 μg loaded lanes. MIC2, GRA7, and TY signals were normalized to the α-tubulin signal in the corresponding total lysate sample.

Supplemental Citations

1. Jimenez, J.L.; Bashir, R. In silico functional and structural characterisation of ferlin proteins by mapping disease-causing mutations and evolutionary information onto three-dimensional models of their c2 domains. *J. Neurol. Sci*. **2007**, *260*, 114–123, doi:10.1016/j.jns.2007.04.016. Available online: https://www.ncbi.nlm.nih.gov/pubmed/17512949
2. Barylyuk, K.; Koreny, L.; Ke, H.; Butterworth, S.; Crook, O.M.; Lassadi, I.; Gupta, V.; Tromer, E.; Mourier, T.; Stevens, T.J.; et al. A Comprehensive Subcellular Atlas of the Toxoplasma Proteome via hyperLOPIT Provides Spatial Context for Protein Functions. *Cell Host Microbe* **2020**, *28*, 752–766.e9, doi:10.1016/j.chom.2020.09.011. Available online: https://www.ncbi.nlm.nih.gov/pubmed/33053376
3. Nadipuram, S.M.; Kim, E.W.; Vashisht, A.A.; Lin, A.H.; Bell, H.N.; Coppens, I.; Wohlschlegel, J.A.; Bradley, P.J. In vivo biotinylation of the toxoplasma parasitophorous vacuole reveals novel dense granule proteins important for parasite growth and pathogenesis. *mBio* **2016**, *7*, doi:10.1128/mBio.00808-16. Available online: https://www.ncbi.nlm.nih.gov/pubmed/27486190
